# Species and genotype diversity of *Plasmodium* in malaria patients from Gabon analysed by next generation sequencing

**DOI:** 10.1186/s12936-017-2044-0

**Published:** 2017-10-03

**Authors:** Albert Lalremruata, Sankarganesh Jeyaraj, Thomas Engleitner, Fanny Joanny, Annika Lang, Sabine Bélard, Ghyslain Mombo-Ngoma, Michael Ramharter, Peter G. Kremsner, Benjamin Mordmüller, Jana Held

**Affiliations:** 1 Institut für Tropenmedizin, Eberhard Karls Universität Tübingen, Wilhelmstraße 27, 72074 Tübingen, Germany; 2 German Centre for Infection Research, partner site Tübingen, Wilhelmstraße 27, 72074 Tübingen, Germany; 3 PSG Institute of Advanced Studies, Coimbatore, 641 004 India; 4 Department of Medicine II, Klinikum rechts der Isar, Technische Universität München, Munich, Germany; 5 Department of Pediatric Pneumology and Immunology, Charité-Universitätsmedizin Berlin, Berlin, Germany; 6 Berlin Institute of Health, Berlin, Germany; 7 Centre de Recherches Médicales de Lambaréné (CERMEL), Lambaréné, Gabon; 8 Department of Medicine I, Division of Infectious Diseases and Tropical Medicine, Medical University of Vienna, Vienna, Austria

**Keywords:** *Plasmodium malariae*, *Plasmodium falciparum*, *Plasmodium ovale wallikeri*, *Plasmodium ovale curtisi*, Next-generation sequencing, Amplicon, Metagenomics

## Abstract

**Background:**

Six *Plasmodium* species are known to naturally infect humans. Mixed species infections occur regularly but morphological discrimination by microscopy is difficult and multiplicity of infection (MOI) can only be evaluated by molecular methods. This study investigated the complexity of *Plasmodium* infections in patients treated for microscopically detected non-falciparum or mixed species malaria in Gabon.

**Methods:**

Ultra-deep sequencing of nucleus (18S rRNA), mitochondrion, and apicoplast encoded genes was used to evaluate *Plasmodium* species diversity and MOI in 46 symptomatic Gabonese patients with microscopically diagnosed non-falciparum or mixed species malaria.

**Results:**

Deep sequencing revealed a large complexity of confections in patients with uncomplicated malaria, both on species and genotype levels. Mixed infections involved up to four parasite species (*Plasmodium falciparum, Plasmodium malariae, Plasmodium ovale curtisi,* and *P. ovale wallikeri*). Multiple genotypes from each species were determined from the asexual 18S rRNA gene. 17 of 46 samples (37%) harboured multiple genotypes of at least one *Plasmodium* species. The number of genotypes per sample (MOI) was highest in *P. malariae* (n = 4), followed by *P. ovale curtisi* (n = 3), *P. ovale wallikeri* (n = 3), and *P. falciparum* (n = 2). The highest combined genotype complexity in samples that contained mixed-species infections was seven.

**Conclusions:**

Ultra-deep sequencing showed an unexpected breadth of *Plasmodium* species and within species diversity in clinical samples. MOI of *P. ovale curtisi*, *P. ovale wallikeri* and *P. malariae* infections were higher than anticipated and contribute significantly to the burden of malaria in Gabon.

**Electronic supplementary material:**

The online version of this article (doi:10.1186/s12936-017-2044-0) contains supplementary material, which is available to authorized users.

## Background

Malaria in humans is caused by six *Plasmodium* species: *Plasmodium falciparum*, *Plasmodium vivax*, *Plasmodium malariae*, *Plasmodium ovale curtisi, P. ovale wallikeri* and *Plasmodium knowlesi* which, although zoonotic, is an important pathogen in humans in several regions of South East Asia [1, 2]. Whereas current research is focused on malaria caused by *P. falciparum* and increasingly also *P. vivax,* only relatively little effort has been dedicated to research on the other human malaria species. Infections with these species usually present with low parasitaemia; they can persist for long periods and sometimes remain asymptomatic. Besides mono-infections with one *Plasmodium* species, mixed infections within one individual occur [3, 4] and interaction between concurrent species—although not well characterized—may play a role in disease progression and outcome [5, 6]. In clinical care, species determination is commonly performed by light microscopy, which has limited sensitivity and specificity. Double, triple or even quadruple infections can be detected more reliably by molecular methods. In addition, presence of distinct genotypes (strains) of the same species cannot be discerned microscopically. An important proportion of naturally occurring infections consists of multiple genotypes and “multiplicity of infection” (MOI) refers to the number of different genotypes of one species infecting a single host [7]. For *P. falciparum*, MOI has been associated with several conditions, including age of the host, clinical severity, and transmission intensity [8]. Determination of within-host diversity may be a useful marker to assess the impact of interventions [9].

Genotyping of polymorphic genes such as merozoite surface proteins (MSPs) and glutamate-rich protein (*glurp*), by nested-PCR is a gold standard method for assessing MOI in *P. falciparum* infections. An alternative approach uses next generation sequencing (NGS) technologies, which allow high-resolution analyses of a heterogeneous mixture of the parasites within the host [10]. Compared to the standard method, NGS revealed an up to six times higher MOI of *P. falciparum* in a previous study [11]. By generating multiple reads per sample (usually between 100 and 10,000), this technique is very sensitive and able to detect minor alleles. Recent analysis of the conserved cytochrome b gene (*cytb*) using NGS, detected multiple genotypes infections (up to 4) including non-falciparum species in 10% of 437 samples collected in Cameroon [12].

Here, a metagenomics sequencing based approach was applied using three pan-*Plasmodium* primer sets for nucleus (18S rRNA), mitochondrion (*cytb*), and apicoplast (*clpC*) encoded genes to characterize the different *Plasmodium* species that infect patients with microscopically diagnosed non-falciparum malaria in the department of Tsamba-Magotsi, Gabon, a remote area of rural Central Africa [13]. By this approach, parasite diversity in 46 Gabonese symptomatic patients is described, that are sampled over a time period of 3 years and provide a baseline for research on the epidemiology and potential influence of non-falciparum malaria on disease burden in Central Africa.

## Methods

### Patients

Parasite DNA for this study was extracted from dried blood spots on filter paper of 46 patients with uncomplicated, microscopically diagnosed non-*falciparum* or mixed species malaria, which represents a minority of malaria cases in the region but was found in more than 20% in a cross-sectional survey in the area [13]. Blood samples from two clinical studies were used. From the first study (recruited 2008–2010) samples of 30 patients were included, details of patients’ characteristics are published elsewhere [14]. Briefly, patients with uncomplicated malaria, defined as symptoms and presence of *P. ovale* or *P. malariae* in thick blood smear, either as mono or mixed infection were included after informed consent was given. All patients received artemether-lumefantrine as anti-malarial chemotherapy and recovered from the infection. From the second study (recruited 2012–2013) 16 samples of patients that were included for molecular assessment of *Plasmodium* spp. were used. Both studies were approved by the regional ethics committee (Comité d’Ethique Régional Indépendant de Lambaréné) and followed the principles of the Declaration of Helsinki in its 5th revision.

### Amplification and 454 sequencing

Capillary blood collected on filter paper from patients prior to anti-malarial treatment was processed for DNA extraction using QIAamp DNA blood mini kit (Qiagen) according to the manufacturer’s specifications. For malaria species and genotype identification using NGS, we designed three *Plasmodium* genus-specific primer sets from the conserved region flanking the highly polymorphic nucleotide sequence of the 18S rRNA, the mitochondrial cytochrome b (*cytb)* and the apicoplast caseinolytic protease C gene (*clpC*). All primers were 5′-fused to universal tail sequences. Using 2.5 µl of DNA extract, target specific PCR was carried out by using Phusion High-Fidelity PCR master mix (Finnzymes) for 35 cycles. The 454 MID kit (Multiplicom) was used to perform subsequent PCR addition of a 454-adaptor sequence linked to multiplex identifiers (MID) in order to discriminate the patients in following analysis steps. Here, the 100 times diluted first round PCR product was used as template and amplified for 20 cycles. All reactions were carried out using a Biometra T2 professional Thermocycler. The corresponding primer sequences and annealing temperature are given in Table 1. PCR amplicons were purified using AmPure XP kit (Agencourt) according to standard procedures (Roche Technical Bulletin No. 2011-007). Quality and purity of amplicons were checked using the Agilent DNA 1000 assay kit on a 2100 Bioanalyzer (Agilent Technology) and subsequently quantified using the Quant-iT Picogreen dsDNA reagent (Invitrogen) on a Fluoroskan Ascent microplate Fluorometer (ThermoScientific). Based on individual DNA concentration, each amplicon was diluted to 10^7^ molecules/µl stock solutions. Amplicons were pooled in equimolar concentration to generate a single library and further processed following the GS Junior emPCR LibA method (Version April 2011) for emulsion PCR (emPCR) using a low copy per bead ratio (0.25 cpb). 500,000 DNA enriched beads were loaded onto a GS Junior Picotiter plate following the GS Junior sequencing manual (Version April 2011) and sequencing was performed in both, forward and reverse direction using the GS Junior Titanium sequencing kit.

**Table 1:** Primers used in this study

Primer name	5′–3′ sequence^a^	Annealing (°C)
SSU-Fwd	AAGACTCGGCAGCATCTCCAGTGAAATTCTTAGATTTTCTG	58
SSU-Rev	GCGATCGTCACTGTTCTCCACGTGTTGAGTCAAATTAAGC	
Cyt-Fwd	AAGACTCGGCAGCATCTCCAGAGTGGATGGTGTTTTAGAT	58
Cyt-Rev	GCGATCGTCACTGTTCTCCAGTGCTACCATGTAAATGTAA	
Clpc-Fwd	AAGACTCGGCAGCATCTCCAGGTCAATTAACAGAACAA	55
Clpc-Rev	GCGATCGTCACTGTTCTCCATAGTTAATCTATTTAATAATTC	
rPLU6^b^	TTAAAATTGTTGCAGTTAAAACG	
rPLU5^b^	CCTGTTGTTGCCTTAAACTTC	
p-AVL-F	GGAATGACAATGTCGTAAAACAAAGTAT	
P-AVL-R	ATACTTTGTTTTACGACATTGTCATTCC	

^a^Underlined region represents universal tag sequences

^b^Snounou et al. [25]

### Sequence analysis

**Fig. 1:**
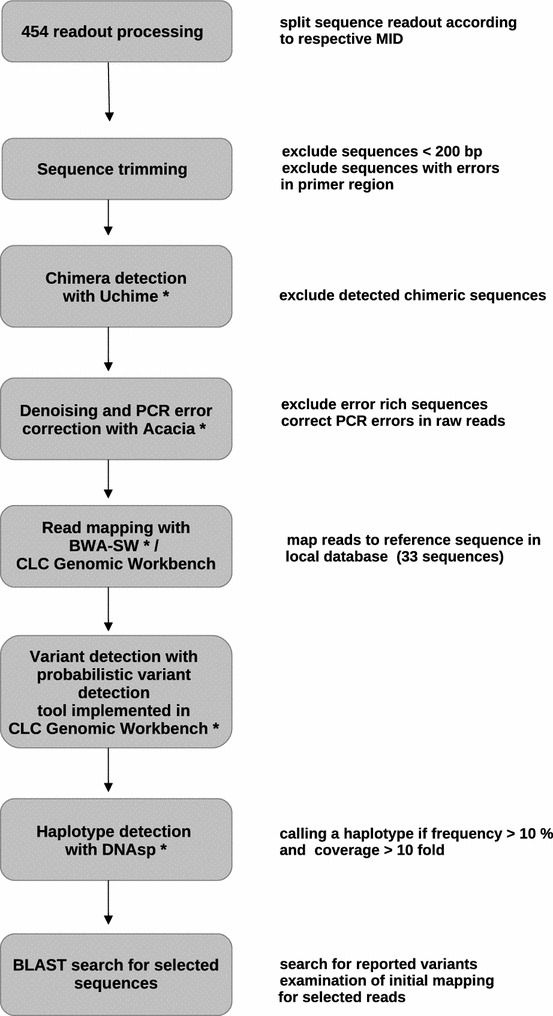
Bioinformatics pipeline. Flowchart describing bioinformatic pipeline. The pipeline is divided into a quality control part, mapping and variant calling part. * used with default settings

Figure 1 summarizes the bioinformatics pipeline used to process *Plasmodium* sequence reads generated by GS Junior. The *sfffile* program (SFF Tools, Roche) was used to split raw sequence data based on multiplex identifier (MID). Low quality and short reads (< 200 bp) were excluded prior to analysis. Sequencing errors (PCR noise) and homopolymer stretches were corrected with Acacia [15]. Chimeric sequences were detected by using the software Uchime [16] and excluded from further analysis. Both programs were run with default parameters. High-quality filtered reads were mapped to a local reference database comprising 18S rRNA, *cytb* and *clpC* gene sequences of *Plasmodium* spp. downloaded from GenBank (Table 2). Unmapped sequences were further analyzed using BLAST searches against the NCBI nucleotide database and the *Plasmodium* database (Plasmodb) [17]. Single nucleotide polymorphisms (SNPs) in the analyzed genes were determined using the probabilistic variant detection method-implemented in the CLC Genomics Workbench 5 (CLC Bio, Aarhus, Denmark). Haplotypes were determined with DnaSP [18] using the following thresholds: SNP frequency equal or greater than 10% as well as per gene read coverage ≥ 10 fold. *Plasmodium* species genotypes were identified by querying consensus sequences against the NCBI GenBank database. All SNP positions were reported as absolute positions in the best matching NCBI reference sequence. Polyclonal infections were identified based on SNPs in the variable region (V5) of the 18S gene, giving the number of genotypes infecting one host (referred to as MOI). The number of genotypes obtained by this marker typically leads to an underestimation of polyclonality and characterization of genotypes based on more polymorphic genes might better estimate the scale of multiple infections. However, this would make comparisons between species more difficult as different genes would be evaluated.

**Table 2:** *Plasmodium* spp. reference sequences used for 454-reads mapping

Plasmodium species	GenBank Acc.
*Plasmodium falciparum* 18S ribosomal RNA gene (S-type)	HQ283222.1
*Plasmodium falciparum* 18S ribosomal RNA gene (A-type)	JQ627152.1
*Plasmodium vivax* 18S ribosomal RNA gene (A-Type)	JQ627158.1
*Plasmodium vivax* 18S ribosomal RNA gene (O-Type)	U93235.1
*Plasmodium vivax* 18S ribosomal RNA gene (S-Type)	U93234.1
*Plasmodium berghei* 18S ribosomal RNA gene	M19712.1
*Plasmodium berghei* 18S ribosomal RNA gene	M14599.1
*Plasmodium malariae* 18S ribosomal RNA gene	M54897.1
*Plasmodium ovale* 18S ribosomal RNA gene	AB182493.1
*Plasmodium ovale* 18S ribosomal RNA gene	AB182489.1
*Plasmodium yoelii* 18S ribosomal RNA gene	AF180727
*Plasmodium knowlesi* 18S ribosomal RNA gene	JF714686.1
*Plasmodium cf. inui* 18S ribosomal RNA gene	FJ619091.1
*Plasmodium simiovale* 18S ribosomal RNA gene	AB287287.1
*Plasmodium ovale wallikeri* cytochrome b gene	KJ930413.1
*Plasmodium ovale curtisi* cytochrome b gene	KP050432.1
*Plasmodium malariae* cytochrome b gene	LT594637
*Plasmodium vivax* cytochrome b gene	JN788776
*Plasmodium falciparum* cytochrome b gene	KC175316.1
*Plasmodium berghei* cytochrome b gene	DQ414645.1
*Plasmodium knowlesi* cytochrome b gene	JQ345523.1
*Plasmodium* spp. from Gabon cytochrome b gene	AF069623.1
*Plasmodium yoelii* cytochrome b gene	DQ414658.1
*Plasmodium falciparum* HB3 clpC gene for caseinolytic protease C	DQ642846.1
*Plasmodium ovale* curtisi clpC gene for caseinolytic protease C	KP050446.1
*Plasmodium ovale* wallikeri clpC gene for caseinolytic protease C	KP050439
*Plasmodium malariae* clpC gene for caseinolytic protease C	AB649418.1
*Plasmodium vivax* clpC gene for caseinolytic protease C	AB471871.1
*Plasmodium gaboni* clpC gene for caseinolytic protease C	HQ842630.1
*Plasmodium knowlesi* clpCgene for caseinolytic protease C	AB471880.1
*Plasmodium simiovale* clpCgene for caseinolytic protease C	AB471881.1
*Plasmodium cf. ovale* clpC gene for caseinolytic protease C	HQ842632.1
*Plasmodium yoelii* clpC gene for caseinolytic protease C	DQ417625.1
*Plasmodium berghei* clpC gene for caseinolytic protease C	AB649421.1

## Results

### Sequence statistics from patients

The 46 patient samples were analyzed in batches of four sequencing runs on a Roche 454 GS Junior sequencer. The median number of raw reads assigned to each sample was 3829 (range 1563–11,590). However, after removal of chimeric and low-quality reads, the final informative sequences available for each sample was 3165 (range 655–9091). The sequence coverage for each locus per sample is reported in the Additional file 1. Differences in coverage between loci and overall batch to batch variation in the number of raw reads were observed. However, there is no evidence that this substantially influences the results, as the overall coverage per sample is expected to be high enough to detect minor genotypes.

### Species discrimination by deep sequencing

Each filtered read was mapped to the target genes of the reference species for identification. Mixed-species malaria infections identified by the metagenomics approach were also detected by conventional nested-PCR in combination with gel electrophoresis [14]. By NGS, it was possible to achieve a more detailed resolution of the species diversity that included identification of nine novel genotypes and polyclonal infections using the same gene in all six investigated *Plasmodium* spp.

A minimum of three high quality reads had to be assigned to at least one of the target loci to be included in the analysis. There were 44 (96%) patients with *P. falciparum,* 15 (33%) with *P. malariae*, 7 (15%) with *P. ovale curtisi*, and 8 (17%) with *P. ovale wallikeri* infections. Different parasite combinations in mixed infections and up to four species in the same host were detected. Of 46 samples, 24 contained only *P. falciparum*, another two contained only *P. malariae* or *P. ovale wallikeri*. The remaining samples contained double (n = 15), triple (n = 2), and quadruple (n = 3) *Plasmodium* species infections (Table 3).

**Table 3:** Number of *Plasmodium* spp. infections detected by deep sequencing

Species	Infection type	Patients (n)
*Pfal*	Mono infection	24
*Pmal*	Mono infection	1
*Pow*	Mono infection	1
*Pfal*, *Pmal*	Double infection	10
*Pfal*, *Poc*	Double infection	3
*Pfal*, *Pow*	Double infection	2
*Pfal*, *Pmal*, *Pow*	Triple infection	1
*Pfal*, *Poc*, *Pow*	Triple infection	1
*Pfal*, *Pmal*, *Poc*, *Pow*	Quadruple infection	3

Pfal, *P. falciparum*; Pmal, *P. malariae*; Poc, *P. ovale curtisi*; Pow, *P. ovale wallikeri*

### *Plasmodium falciparum* infections

*Plasmodium falciparum* specific sequence reads were identified in all but 2 (96%) of the study samples. Based on the 18S A-type gene sequence, four novel genotypes (GenBank KJ170098—KJ170101) were identified, all are a single nucleotide polymorphism when compared to the reference sequence (GenBank JF681166) as shown in Table 4. No other sequences with these SNPs were found when a similarity search against GenBank was performed. Co-infections with other species were found in 20 patients (45%).

**Table 4:** Polymorphic Nucleotide positions compared to GenBank best-hits reference sequences

Genotypes	Origin	386	407	504	517	529		
*P. falciparum* (JF681166)	China	G	T	G	A	C		
*P. falciparum* (KJ170098)	Gabon	A	C	.	–	.		
*P. falciparum* (KJ170099)	Gabon	.	.	.	.	G		
*P. falciparum* (KJ170100)	Gabon	A	.	.	.	.		
*P. falciparum* (KJ170101)	Gabon	.	.	A	.	.		

		1036	1189					
*P. malariae* (AF488000)	Myanmar	G	–					
*P. malariae* (KJ170105)	Gabon	.	T					
*P. malariae* (KJ170106)	Gabon	A	T					

		1005	1026	1109	1112	1128	1131	1132
*P. ovale wallikeri* (AB182493)	Indonesia	A	C	G	G	A	G	A
*P. ovale wallikeri* (KJ170102)	Gabon	.	.	.	.	G	.	.
*P. ovale wallikeri* (KJ170103)	Gabon	G	T	A	A		A	G
*P. ovale wallikeri* (KJ170104)	Gabon	G	T	.	.	.	.	.

Each dot represents nucleotide similarity with the GenBank reference sequences. Dashes represent deletions. Numbers at the column header represent nucleotide positions corresponding to GenBank reference sequences

### *Plasmodium malariae* infections

A total of 15 infections were observed with the quartan malaria parasite, one as mono and the remaining ones as co-infection with other species. Based on the 18S rRNA gene, the parasites were further characterized as *P. malariae*-Asian type 1 (n = 1) and *P. malariae*-Asian type 2 (n = 14) [19]. Two unique genotypes (GenBank KJ170105, KJ170106) similar to *Plasmodium cf. malariae* type 2 were obtained (GenBank AF488000, 99% identity). Each genotype is defined by either a single nucleotide substitution or insertion compared to *P. malariae*-Asian type 2 (Table 4) (see comments below).

### *Plasmodium ovale wallikeri* and *P. ovale curtisi* infections

*Plasmodium ovale wallikeri* and *P. ovale curtisi* were identified in the dataset supported by reads matching to specific 18S, *cytb,* or *clpC* sequences of the two *P. ovale* species (Additional file 1). In addition, mixed infections of *P. ovale curtisi* and *P. ovale wallikeri* were observed in four patients (Table 3).

All of *P. ovale curtisi* genotypes identified from the samples were similar to previously submitted sequences. Three novel genotypes of *P. ovale wallikeri* were identified based on 18S rRNA gene sequence polymorphisms (GenBank KJ170102—KJ170104). One of them possesses a single nucleotide substitution at position 1128. Genotype 2 and Genotype 3 showed six and two nucleotide substitutions when compared to the corresponding reference sequence (GenBank AB182493), respectively (Table 4).

### Detection of *P. ovale curtisi* 18S rRNA gene variant

**Fig. 2:**
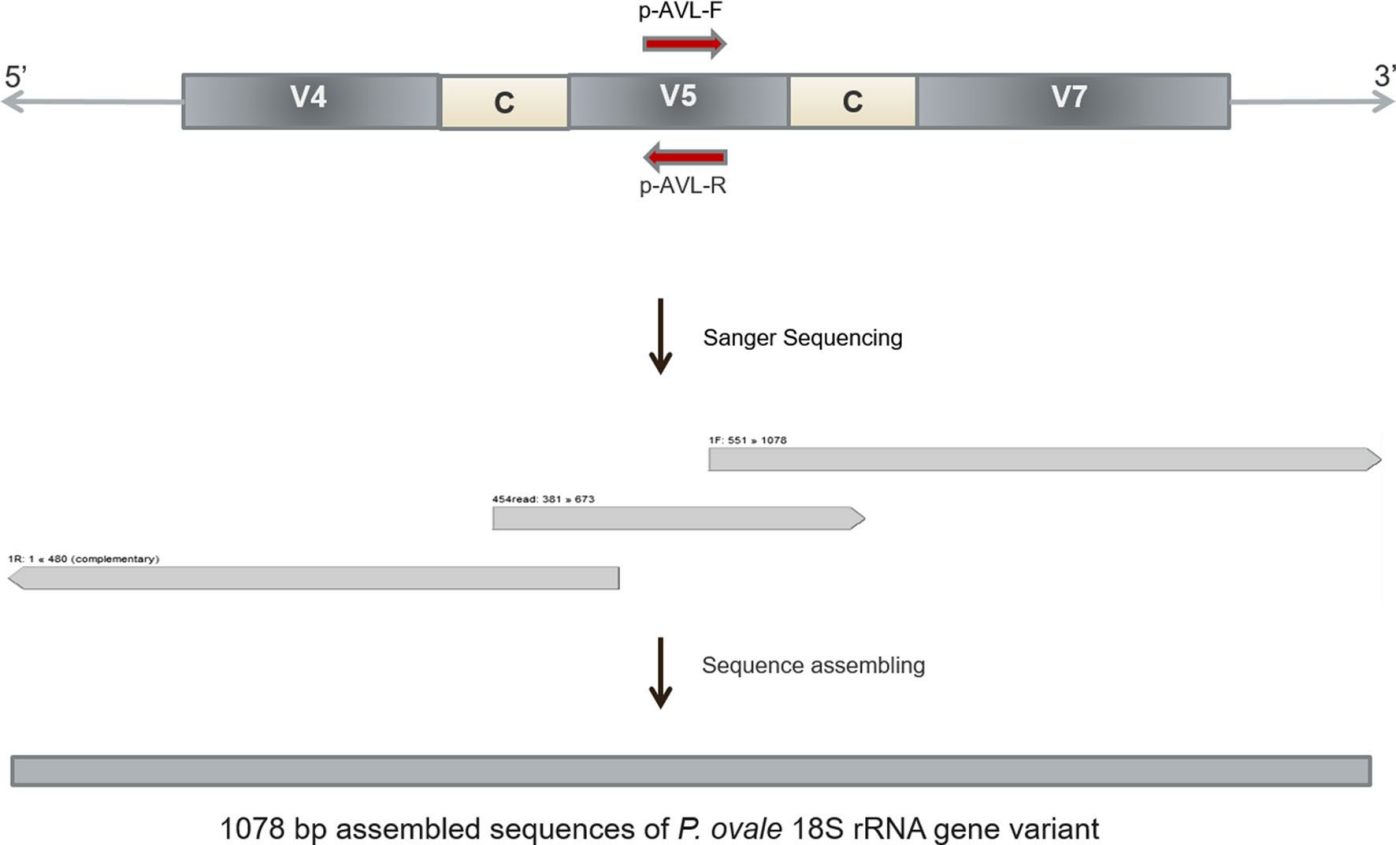
Schematic representation of the sequencing approach by Sanger method to determine the new 18S rRNA gene type sequence of *P. ovale* spp. Conserved pan-*Plasmodium* 18S primers (rPLU6 and rPLU5) were first used to amplify the gene spanning three variable regions and two conserved blocks. The nucleotide sequences upstream and downstream of the 454 sequencing target region (V5) of the gene were determined by direct sequencing of the initial product using p-AVL-F and p-AVL-R primers. Partial 18S gene (1078 bp length) was obtained by assembling 454 reads with the sequences obtained using p-AVL-F and p-AVL-R primers

In three of the samples a fraction of reads (range 10–15 reads) could not be mapped to any reference sequence. A sequence identity search against the NCBI database initially did not show significant similarity with respect to query coverage and maximum identity with any of the *Plasmodium* spp. 18S sequence. A 1078 bp length sequence was constructed (GenBank KJ170108), spanning the variable regions 4 and 7 along with the 454 amplicon target (V5) by PCR and Sanger sequencing with pan-*Plasmodium* and specific primers, respectively (Fig. 2). The generated sequence showed 100% similarity to the draft genome of *P. ovale* (*P. ovale* Blast Server, Sanger Institute) and a recently discovered 18S rRNA gene variant (GenBank KF696378) of *P. ovale curtisi* [20].

### Multiplicity of infection

**Fig. 3:**
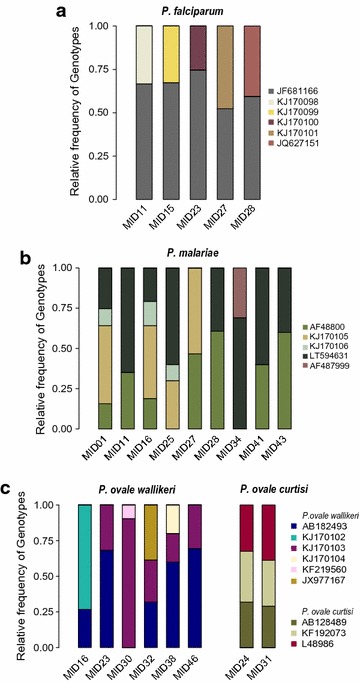
Relative frequency of each *Plasmodium* spp. genotype within one infected host. **a** Relative frequency of *P. falciparum* genotypes (defined by the 18S A-type gene variation); **b** relative frequency of *P. malariae* genotypes. **c** Relative frequency of *P. ovale curtisi* and *P. ovale wallikeri*

An overview of the MOI based on the variable region (V5) of the 18S gene for the different *Plasmodium* species is given in Table 5. The relative frequency of each genotype per sample based on the number of reads is plotted in Fig. 3. Multiple *P. falciparum* genotype (MOI) infections were detected in five patients with a maximum of two genotypes per sample. All *P. falciparum* monoinfections carried a single genotype, except one patient (MID15) who carried two genotypes. Multiple genotype infections were found in two of the seven *P. ovale curtisi* infected samples (MOI of 3). Among eight samples infected with *P. ovale wallikeri*, six harboured multiple *P. ovale wallikeri* genotypes (MOI of 2–3). Analysis of the *cytb* and *clpC* reads showed 100% similarity to the published reference sequences. Nine samples contained multiple *P. malariae* genotypes (MOI of 2–4). The number of different genotypes per sample was greater for *P. malariae* when compared to the other species, showing up to four genotypes within one sample.

**Table 5:** Multiplicity of *Plasmodium* spp. infections

Sample id	Infecting species	Number of genotypes for each species				
		*Pfal*	*Pmal*	*Poc*	*Pow*	Total (MOI)
MID01	*Pmal*		4			4
MID11	*Pfal, Pmal*	2	2			4
MID15	*Pfal*	2				2
MID16	*Pfal, Pmal, Poc, Pow*	1	4	na	2	7
MID23	*Pfal, Poc, Pow*	2			2	4
MID24	*Pfal, Poc*	1		3		4
MID25	*Pfal, Pmal*	1	3			4
MID27	*Pfal, Pmal*	2	2			4
MID28	*Pfal, Pmal, Poc, Pow*	2	2	1	1	6
MID30	*Pfal, Pmal, Pow*	1	na		2	3
MID31	*Pfal, Poc*	1		3		4
MID32	*Pfal, Pow*	1			3	4
MID34	*Pfal, Pmal*	1	2			3
MID38	*Pfal, Pow*	1			3	4
MID41	*Pfal, Pmal*	1	2			3
MID43	*Pfal, Pmal*	1	2			3
MID46	*Pow*				2	2

Pfal, *P. falciparum*; Pmal, *P. malariae*; Poc, *P. ovale curtisi*; Pow, *P. ovale wallikeri*; na, no 18S sequence obtained

Reads mapping to *cytb* and *clpC* showed 100% sequence identity to the reference sequences for all species (KC175316 and DQ642846 for *P. falciparum*; KJ930413 and KP050439 for *P. ovale wallikeri*; KP050432, KP050446 for *P. ovale curtisi*; HQ842634 and AB649418 for *P. malariae*).

## Discussion

Gabon is highly endemic for malaria with perennial transmission of *P. falciparum* and co-endemicity of *P. malariae* and *P. ovale* [21, 22]. Gabon has a low population density (6.7 persons per square km in 2015) [23], but population mobility is high, and a large fraction of inhabitants live close to or within non-cultivated areas, that contain diverse and intact wildlife [24]. Originally, the study was designed to assess the efficacy of artemisinin combination therapy (artemether-lumefantrine) in uncomplicated non-*falciparum* and mixed species malaria [14]. Species identification was based on microscopy and verified by species-specific nested PCR [25]. Here, a metagenomics approach was designed based on high throughput sequencing to re-analyze the diversity of *Plasmodium* spp. Two studies were conducted recently using NGS to explore the evidence of zoonotic transmission of *Plasmodium* spp. in humans [12, 26]. These studies were designed to differentiate *Laverania* species of apes and humans based on the diagnostic single nucleotide polymorphism found in the mitochondrial DNA (mtDNA). The current study expanded the target genes to three genes including the 18S rRNA and *clpC* gene to improve accuracy and assess the diversity of *Plasmodium* populations in blood samples of malaria patients. The 18S rRNA gene is one of the most commonly used targets in the molecular diagnosis of *Plasmodium* spp. including a widely used nested PCR and a number of real-time PCR assays [25, 27]. Due to the high copy numbers (5–10 copies), and the presence of both highly conserved and variable regions, 18S genes are well-suited and frequently used molecular targets for the detection and discrimination of *Plasmodium* species. Until to date, no study has applied these genes to identify *Plasmodium* spp. using NGS.

As expected, results obtained by conventional species-specific PCR were reproduced upon DNA re-extraction and analysis by deep sequencing. Beyond this, this analysis gives a snapshot in time; showing the breadth of co-infections and an unbiased estimate of infection intensity of the different *Plasmodium* spp. in a cohort of patients with uncomplicated malaria. *P. malariae* is widespread in Africa but rarely characterized by molecular techniques. Based on morphological differences and sequence polymorphisms compared to genuine *P. malariae* (Uganda CDC isolate), two possible subtypes were identified in Asia: *P. cf. malariae* type 1 and *P. cf. malariae* type 2 [19]. It would be interesting to investigate if the Asian and the Ugandan type differ in their sensitivity to artemisinins, since a reduced sensitivity of an infection in Uganda has been observed [28, 29] but all patients in this study responded well to the treatment [14]. Only few studies have looked at MOI of non-falciparum species. It has been shown in one study that *P. malariae* infections in Malawi, often consisted of multiple genotypes per infected individual and showed a surprisingly similar pattern when compared to *P. falciparum* [30]. Up to five genotypes were detected from a single sample by multilocus genotyping based on microsatellite markers in asymptomatic carriers [31]. There are also up to four different genotypes of *P. malariae* in two patients and three different genotypes of *P. ovale wallikeri* and *P. ovale curtisi* each in two patients in the here presented study based on the 18S region, respectively. Many of the *P. falciparum* infections were on the contrary caused only by one genotype when judged by polymorphisms in the 18S rRNA gene. These results are surprising as one would assume a lower MOI in *P. malariae* and both *P. ovale* species as the reported prevalence in this region is low. However, results are also in line with earlier findings from Cambodia for *P. ovale* [32], reflecting the possibility that these parasite species are more prevalent than previously thought. Evidence suggests that *P. malariae* is not completely species-specific and also prevalent in non-human primates [29, 33], having a larger pool of hosts. In addition, infections with these species might be more chronic and long-lived so that multiple genotypes could accumulate in one host. It has to be considered that the marker used to define a genotype in this study is not as polymorphic as markers commonly used to define genotypes; for example microsatellites, or genes like *msp1*, *msp2* and *glurp* for *P. falciparum* [34]. It is expected that the number of genotypes are larger if more polymorphic markers had been used. The advantage of our approach is that homologous genes between the different species can be compared and one can get an impression on the population structure of the co-infecting species. Particularly, because there is no evidence that selection leads to different mutation rates in this gene region for the different species, this can be one approach to compare diversity between species. These results highlight the potential of amplicon-based high throughput sequencing combined with adequate polymorphic markers to obtain reliable molecular characterization of other non-falciparum species, where only limited data are available. Despite short-read length (< 400 nt) generated by the NGSs technique, significant dimorphism of the targeted genes allowed accurate assignment of reads between *P. ovale curtisi* and *P. ovale wallikeri.* By using a multiple locus approach, co-infections of the two *P. ovale* species in four patients were detected, adding yet further evidence that the two species do not recombine in nature. The primer for the *cytb* gene was selected from a region not including the ape-specific SNPs leading to potential inability to detect non-human species. However, there was no evidence for non-human *Plasmodium* species infections based on the analysis of the other two genes.

Two structurally distinct types of 18S rRNA have been reported in many *Plasmodium* species [35]. In *P. falciparum,* type A and type S has been described with up to 11% difference in the sequences between the two types [36]. The existence of paralogous 18S rRNA genes in *P. malariae*, *P. ovale wallikeri* and *P. ovale curtisi* genome is not well described until now. The new type of 18S rRNA gene sequence from this study, together with the recent report [20], clearly suggests that at least two different forms exist in *P. ovale curtisi* and *P. ovale wallikeri* [20]. A real-time PCR based assay targeting this new sequence showed high sensitivity and specificity and can be used for the differential diagnosis of *P. ovale* species infections (unpublished observation).

The main limitation of techniques that involve PCR is the inherent risk of contamination and the NGS approach is no exception. Thus, an essential aspect of sample preparation for sequencing is the careful adoption of experimental strategies aimed at minimizing cross-contamination. In addition, amplicon-based NGS are prone to errors such as chimeras, a well-known issue in metagenomics analyses of environment bacterial communities. Several measures to minimize and avoid these underlying problems at each step, including a non-template control to check contamination of reagents during each target amplification, a separate work station for DNA extraction, PCR assay set-up, and post-PCR processing are necessary.

The true burden of disease caused by *P. malariae* and the *P. ovale* species is not known as these species are mostly underdiagnosed despite their worldwide distribution [29]. Malaria caused by these parasites is generally more benign when compared to *P. falciparum.* Infections present often with a low level parasitaemia that is difficult to detect by microscopy, but might cause a more chronic illness associated with anaemia [37]. Recent reports reveal a higher than expected prevalence of these species in many African countries when diagnosed by PCR [38], going in line with the deep sequencing result. Currently, deep sequencing methods may not be applicable in field settings but can be helpful in longitudinal epidemiological studies to investigate the emergence and change in composition of plasmodial species. The study population was highly selected and represents only a minority of all malaria cases in the area. Microscopic species differentiation is not reliable, particularly when parasite density is low. Therefore, it is unlikely that the appearance of new plasmodial species is noted without molecular techniques. Diversity of the two *P. ovale* species, and characterization of the *P. malariae* population should be taken into consideration for the design, endpoints and feasibility of malaria control strategies, e.g. longer follow up or separate treatment of hypnozoites. Fortunately, artemisinin combination therapy was efficacious in all patients of the present study but emergence of novel zoonotic species or isolates that may be inherently less responsive to current treatment regimens shall be detected early, to provide a specific treatment regimen, especially when severe malaria can occur, as in the case of *P. knowlesi* infections. The impact on malaria vaccine development shall even be stronger since cross-protection between *Plasmodium* species is limited [39] and mechanisms of high-grade protection are often not known but likely to be different for each parasite species.

## Conclusions

Parasite diversity of *Plasmodium* species in naturally acquired malaria is larger than expected. The population structure, especially of non-falciparum species, needs further assessments to better understand the prevalence and biology of these parasites. Metagenomics analysis by deep sequencing provides a tool for this and boosts the understanding of naturally acquired malaria. It will be exciting to expand such investigations to other malaria-endemic regions and larger cohorts.

## Additional file

**Additional file 1: Table S1.** Number of high-quality 454 reads mapped to *Plasmodium* spp. references.

## Abbreviations

clpC: caseinolytic protease C
cytb: cytochrome b
MOI: multiplicity of infection
mtDNA: mitochondrial DNA
NGS: next-generation sequencing
PCR: polymerase chain reaction
SNPs: single nucleotide polymorphisms
