# Supplementary material for: Species and genotype diversity of Plasmodium in malaria patients from Gabon analysed by next generation sequencing
Source: Malar J. 2017 Oct 3;16:398. doi: 10.1186/s12936-017-2044-0 (PMC5627438; doi:10.1186/s12936-017-2044-0)
Supplement: Supplementary file 1 — Additional file 1: Table S1. Number of high-quality 454 reads mapped to Plasmodium spp. references. [file 12936_2017_2044_MOESM1_ESM.pdf]

Sample ID	*P. falciparum*				*P. malariae*			*P. ovale curtisi*			*P. ovale wallikeri*			Coverage
	18S A	18S S	cytb	clpc	18S	cytb	clpc	18S	cytb	clpc	18S	cytb	clpc	
MID01	0	0	0	0	270	539	79	0	0	0	0	0	0	888
MID02	263	40	313	252	0	0	0	0	0	0	0	0	0	868
MID03	547	94	589	230	0	0	0	0	0	0	0	0	0	1460
MID04	313	96	814	291	0	0	0	0	0	0	0	0	0	1514
MID05	470	10	569	272	0	0	0	0	0	0	0	0	0	1321
MID06	324	80	602	269	0	0	0	0	0	0	0	0	0	1275
MID07	184	162	180	129	0	0	0	0	0	0	0	0	0	655
MID08	0	0	266	699	0	0	0	0	0	0	0	0	0	965
MID09	390	423	611	679	0	0	0	0	0	0	0	0	0	2103
MID10	403	398	572	749	0	0	0	0	0	0	0	0	0	2122
MID11	29	38	32	93	761	402	491	0	0	0	0	0	0	1846
MID12	6309	425	679	645	0	0	0	0	0	0	0	0	0	8058
MID13	326	497	869	702	0	0	0	0	0	0	0	0	0	2394
MID14	278	213	918	704	0	0	0	0	0	0	0	0	0	2113
MID15	221	298	717	2121	0	0	0	0	0	0	0	0	0	3357
MID16	266	84	47	651	84	0	32	0	0	43	19	0	0	1226
MID17	211	567	683	1241	0	0	0	0	0	0	0	0	0	2702
MID18	164	209	623	4293	0	0	0	0	0	0	0	0	0	5289
MID19	314	992	706	1240	0	0	0	0	0	0	0	0	0	3252
MID20	81	460	589	543	0	0	0	0	0	0	0	0	0	1673
MID21	400	245	763	375	0	0	0	0	0	0	0	0	0	1783
MID22	396	312	807	704	0	0	0	0	0	0	0	0	0	2219
MID23	739	223	306	2226	0	0	0	4	0	13	2363	2628	0	8502
MID24	329	347	67	218	0	0	0	1653	2316	2007	0	0	0	6937
MID25	2192	1675	2256	1847	91	0	0	0	0	0	0	0	0	8061
MID26	1244	1151	2924	2895	0	0	0	0	0	0	0	0	0	8214
MID27	145	237	23	163	3006	2002	1682	0	0	0	0	0	0	7258
MID28	1213	636	269	2180	759	1489	291	28	445	0	10	10	0	7330
MID29	253	505	18	273	3402	2323	1910	120	0	0	287	0	0	9091
MID30	2177	1645	2046	3070	0	0	28	0	0	0	26	0	0	8992
MID31	198	104	49	0	0	0	0	939	856	1486	0	0	0	3632
MID32	27	0	0	1604	0	0	0	0	0	0	678	124	8	2441
MID33	28	0	0	0	673	0	1249	0	0	0	0	0	0	1950
MID34	585	220	373	1576	100	383	0	0	0	0	0	0	0	3237
MID35	547	116	86	1996	562	673	44	0	0	0	0	0	0	4024
MID36	821	132	693	1970	0	0	0	0	0	0	0	0	0	3616
MID37	1389	241	1181	1442	0	0	0	0	3	3	0	0	0	4259
MID38	759	423	511	1166	0	0	0	0	0	0	22	21	0	2902
MID39	490	316	843	1736	0	0	0	0	0	0	0	0	0	3385
MID40	329	26	241	2234	402	539	0	0	0	0	0	0	0	3771
MID41	343	38	871	1577	15	81	0	0	0	0	0	0	0	2925
MID42	495	69	631	1581	84	233	0	0	0	0	0	0	0	3093
MID43	829	121	859	3278	10	6	0	0	0	0	0	0	0	5103
MID44	864	41	749	2206	0	0	0	0	0	0	0	0	0	3860
MID45	944	120	179	2012	0	0	0	0	0	0	0	0	0	3255
MID46	0	0	0	0	0	0	0	0	0	0	718	655	1992	3365
